# De Herophili Celeberrimi Medici Vita, Scriptis, atque in Medicina Meritis

**Published:** 1843-01

**Authors:** 


### ART. VIII.

*De Herophili Celeberrimi Medici Vita, Scriptis, atque in Medicina Meritis.* Auctore CAR. FRID. HENR. MARX.—*Gottingæ,* 1840. 4to, pp. 60.

IN a recent Number of our Journal, after expressing some surprise that the name of Professor Marx was not better known in England, we gave some account of his work on Paracelsus, which probably no man in Europe could have written but himself; and if we cannot say quite so much for that which we are now about to notice, we may at any rate safely affirm that no man in Europe could have executed it better. It belongs to a very useful class of monographs, which serve to throw great light upon particular periods of the ancient history of medicine, and which are not uncommon in Germany,* though in England they are almost unknown.† Except the very highest names in ancient medical history, perhaps few are better known in England than that of Herophilus. Probably every one has a vague idea that he was a great anatomist, and recollects meeting with his name, when he was a medical student, in his "Elements of Anatomy" and in Celsus. More than this is not so generally known; and therefore we gladly take the opportunity of giving an analysis of Professor Marx's work, and of thus helping to revive the fame of one who held (according to Fallopius ‡) the same place among the ancients as Vesalius among the moderns, and whose authority in anatomy was equal to that of the Gospel in religion. Before doing this, however, it may be as well to give some slight sketch of the state of anatomy before his time, and to notice the dates and authors of the principal discoveries in that branch of medical science. The earliest improvements seem to be due to the Pythagorean philosopher, Alcmæon, who (in the sixth century before Christ) was the first person who ventured upon systematic dissections,§ instead of resting content with such imperfect glimpses at our internal structure as might be gained from the casual inspection of victims offered in sacrifice, the dressing of wounds, &c. Whether he merely confined himself to the dissection of animals, or whether he so far anticipated the improvements of a later age as to dissect human bodies is uncertain; || but we have reason to believe that the ***Eustachian tube*** was first observed

* Of these we may mention two or three of the best, as they may, perhaps, be new to some of our readers: "Asclepiadis Bithyni Fragmenta digessit et curavit Christ. Gottl. Gumpert, præfatus est Christ. Gothfr. Gruner," Vinariæ, 1794, 8vo; "Antylli, veteris chirurgi, τὰ λείψανα ventilanda exhibet Panagiota Nicolaides præside Curtio Sprengel," Halæ, 1799, 4to; C. G. Kühn, "De Praxagora Coo," in the second volume of his *Opuscula Academica Medica et Philologica,* Lips. 1827-8, 2 vols. 8vo.

† The only English work of this kind that we are aware of is Dr. Milward's *Trallianus Reviviscens,* &c. Lond. 1734, 8vo.

‡ *De Mater. Medicin. in lib. i. Dioscor.* c. 1, tom. i. p. 25, ed. Francof. 1600. See, also, *De Part. Simil.* c. 12, 13. tom. ii. p. 129, 132.

§ Chalcidius, *Comment. in Platon.* "*Timæum,*" p. 340. ed. Meurs.

|| The word *exsectio,* used by Chalcidius, seems to be equally applicable to human and comparative anatomy, implying merely *dissection* generally, that is, as it was usually carried on in the time of the writer. If this be true, *exsectio* will here certainly imply the dissection of animals; and, indeed, we might naturally expect some more precise and positive expression, if the human body were meant. Alcmæon's Pythagorean prejudices would be equally shocked by the touch of the dead body of a man or an animal.

by him,* and that in physiology he advanced far beyond his predecessors. The cochlea of the ear,† and the amnios of the fœtus,‡ owe their names to Empedocles, of Agrigentum, who probably first described them, in the fifth century before Christ. Omitting Anaxagoras and Democritus, whose anatomical labours and speculations furnished no results of sufficient importance to be particularly noticed here, we next come to Hippocrates, the amount of whose knowledge in this (or indeed any other) branch of medical science, it is not easy to determine. For, supposing the preliminary question, "Did Hippocrates ever really exist?"§ to be satisfactorily answered in the affirmative, we find ourselves at once involved in the much more difficult inquiry as to which, of all the numerous treatises that go under his name, were really written by him. These difficulties, however, in the present instance, need not occupy our attention, as we are not aware of any great anatomical or physiological discovery connected with the name of Hippocrates, and this is certainly one of the least valuable parts of the Hippocratic collection. The nerves seem to have been first distinguished from the tendons by Aristotle,‖ who also affirmed that the brain of man is larger than that of any other animal,¶ and who was the first person who restricted the term *aorta* to the great artery of the body.** Praxagoras, the tutor of Herophilus, is generally supposed to have been the earliest author who distinguished between the arteries and veins, though the honour of this discovery is doubted by Kühn.†† During all this time (and indeed much later) the greatest laxity and confusion prevailed in anatomical language, which of course greatly adds to the difficulty of determining the exact amount of knowledge possessed by the ancients in this branch of medical science. A few instances will be sufficient to prove this. The word φλὲψ was originally applied to both the veins and arteries,‡‡ the latter being sometimes called φλέβες σφύζουσαι, or *pulsating veins;* on the other hand, ἀρτηρία in the oldest writings always means the *trachea;* and when in process of time the word was used in a more extended sense, so as to include the *arteries*, the former was called τραχεῖα ἀρτηρία, or *the rough artery*, while the latter were distinguished by the epithet λεῖαι, and were called *the smooth arteries.* Again, not only before the time of Aristotle, but even after the distinction

* This we learn, curiously enough, from Aristotle, who says (*Hist. Anim.* i. 9. § 1,) that Alcmæon was wrong in affirming that goats breathe through their ears. This strange assertion can only be accounted for by supposing that he had observed the canal leading from the anterior and inner part of the tympanum to the fauces; and, if we imagine that, in the animal which he dissected, the membrana tympani had been accidentally destroyed, his mistake may be easily and naturally explained.

† Κοχλιώδης χόνδρος. Plutarch. *De Phys. Philosoph. Decretis,* lib. iv. c. 16.

‡ Julius Pollux, *Onomast.* lib. ii. § 223; Rufus Ephes. *De Hum. Corp. Part. Appellat.* p. 45. ed. Clinch.

§ M. Houdart (p. 23) commences his *Etudes sur Hippocrate* by this question, which, however, he confesses to be "la plus complète des absurdités."

‖ He calls the nerves πόροι. *Hist. Anim.* lib. iv. c. 8. § 2, *et passim.*

¶ *Hist. Anim.* lib. i. c. 13. § 2.

** *Hist. Anim.* lib. i. c. 14, § 3; iii. 3, § 1. Before his time the word was applied both to the other arteries of the body indiscriminately (Rufus Ephes. *De Corp. Hum. Part. Appellat.* p. 42), and also to the bronchia (Ruf. Eph. *ibid.* p. 37; Julius Pollux, *Onomast.* lib. ii. c. 4, § 205; Hippocr. *De Locis in Hom.* tom. ii. pp. 123, 124. ed. Kühn).

†† *Comment. de Prax. Coo,* in his *Opusc. Academ. Med. et Philolog.* tom. ii. p. 136, seq.

‡‡ See the notes on p. 106, l. 14, and p. 257, l. 1, of the Oxford edition of Theophilus *De Corp. Hum. Fabrica.*

between the *nerves* and *tendons* had been established, the word *νεῦρον* continued to be used in a very vague and indefinite way, so much so that in later times it was defined to mean a white, dense body,* of which there are three species, viz., those arising from the brain and spinal marrow, which are properly called *nerves;* those from the muscles, or *tendons;* and those from the bones, or *ligaments.* Several other instances might be mentioned in which the sense given by modern authors to Greek and Latin anatomical terms (and the remark might be extended, though in a less degree, to other branches of medical science,) is so different from the meaning of the words as used by the Greek and Latin writers themselves, that any one, who was not aware of this, would be constantly liable to be misled, and to attribute to the ancients ignorance and blunders of which they are wholly innocent. After these few remarks on the state of anatomical science and language before the time of Herophilus, we may now proceed to give an analysis of Professor Marx's life of that celebrated man.

We do not possess any detailed ancient account of Herophilus, and must therefore be content to glean all our information respecting him from such stray hints and expressions as we can meet with. He appears to have been born at Chalcedon, in Bithynia,† and to have lived in the time of Ptolemy Soter, B. C. 323-283, though the exact year both of his birth and his death is unknown. He was one of the pupils of Praxagoras, and afterwards settled at Alexandria, which city had been lately founded by Alexander, and was rapidly rising into eminence under the enlightened government of the first Ptolemy. Here he soon acquired a great reputation, and was one of the first founders of the medical school in that city, which afterwards eclipsed in celebrity all the others, so much so that in the fourth century after Christ the very fact of a physician's having studied at Alexandria was considered to be a sufficient guarantee of his ability. (Marx, p. 8.) Connected with his residence here, there is rather an amusing anecdote of his proving to a Sophist the possibility of motion, in a way probably more convincing than even Aldrich's famous "solvitur ambulando!" It appears that the philosopher used to deny the existence of motion, and to support his assertion by the following dilemma,—the fallacy of which, by the way, it is much easier to see at once than to explain:‡ "If matter moves, it is either in the place where it is, or in the place where it is not: but it cannot move in the place where it is, and certainly not in the place where it is not; therefore it cannot move at all." He happened, however, to dislocate his shoulder, and sent for Herophilus to replace it, who first began by proving by his own argument that it was quite impossible that any luxation could have taken place; upon which the unhappy sophist begged him to leave such quibbling for the present, and to proceed at once to his surgical treatment. (Marx, p. 6.) He seems to have given his chief attention to anatomy, which he studied not

* Pseudo-Galen, *Defin. Med.* c. 76, tom. xix. p. 366. ed. Kühn. See the note on p. 204, l. 5, of Theoph. *De Corp. Hum. Fabr.*

† His biographers "vie with each other" (as Porson would say,) in correcting and explaining the mistake in the text of Galen, *De Usu Part. Corp. Hum.* lib. i. c. 8, tom. iii. p. 21, where Καρχηδονίῳ is found instead of Χαλκηδονίῳ. Dr. Marx has committed a slight oversight in his quotation from Strabo, where he says that Chalcedon was *Μαγνησίων κτίσμα*: it should be *Μεγαρέων*.—Isensee says (*Gesch. der Med.* p. 87,) that Herophilus "ward zu den letzten Sprösslingen der Asclepiaden gerechnet;" but we do not know where he finds this, except in the *Biographie Médicale.*

‡ See Whately's *Logic*, pp. 371, 372.

merely from the dissection of animals, but from that of human bodies, as is expressly asserted by Galen. (Marx, p. 25.) The famous story of his dissecting criminals alive it seems difficult to disbelieve, though most of his biographers have tried to explain it away, or to throw discredit on it;* for (not to lay much stress on the evident exaggeration of Tertullian, who says that he dissected as many as six hundred,) it is mentioned by Celsus quite as a well-known fact, and without the least suspicion as to its truth; added to which, it should be remembered that such a proceeding would not be nearly so shocking to men's feelings two thousand years ago as it would be at present.† He was the author of several medical and anatomical works, of which nothing but the titles and a few fragments remain. Almost all of these have been collected by Professor Marx with remarkable diligence and accuracy, so that he has left us little to do except to make a few additions (chiefly from Greek authors published since the first appearance of his work in German, 1836), and to correct one or two errors, in doing which we would beg our readers to remember (as we hope to bear in mind ourselves,) Galen's most excellent confession, that "it is difficult for a mere man to avoid making many blunders, some from sheer ignorance, others from a fault of judgment, and others from carelessness."‡ The first work attributed to Herophilus by Professor Marx is entitled Περὶ Αἰτιῶν, *De Causis*, which is done on the authority of a manuscript of Apollonius Citiensis, quoted by Cocchi in his *Discorso dell' Anatomia*, Firenze, 1745, 4to, p. 80. Cocchi's work we have never seen,§ and have therefore been unable to verify the reference; but if (as we are pretty sure,) the passage referred to be the same with that which is afterwards quoted (p. 58,) from Dietz's *Scholia in Hippocr. et Gal.* tom. i. p. 34, it does not seem quite clear to us that the work *De Causis* was written by Herophilus. The passage stands thus: *Θαυμάζω δὲ ἐπὶ τοῖς τὴν πολυθρύλλητον ἀνατομὴν ἐναγκαλιζομένοις Ἡροφιλείοις, μάλιστα δὲ ἐπὶ Ἡγήτορος· ἐν γὰρ τῷ Περὶ Αἰτιῶν περὶ μηροῦ ἐξαρθρήσεως οὕτως ἐμέμνητο τὰ ὑποτεταγμένα διασαφῶν·* which Professor Marx translates as follows: "Apollonius initio inquit, se mirari Herophileos, percelebratam anatomen amplexantibus, maxime vero ipsorum ducem, qui libro *De Caussis* de femore

* Perhaps the strongest argument against the truth of the story is derived from the silence of Galen, who so expressly mentions his dissecting human bodies. It is curious too, that the same accusation is brought against Archigenes, and Galen himself, by one of the later commentators on Hippocrates, (Joannes Alexandrinus, in Dietz's *Scholia in Hippocr. et Gal.* tom. ii. p. 216,) from which it would almost seem that the story was applied to Herophilus, Erasistratus, Archigenes, or Galen, quite at random; for without stronger and more positive evidence, we are hardly called upon to believe it of them all. It may be noticed that Galen's lost work, Περὶ τῆς ἐπὶ τῶν Ζώντων Ἀνατομῆς, *De Vivorum Dissectione*, (Galen. *De Ordine Librorum suorum*, tom. xix. p. 55,) related to the vivisection of animals, and not of man.

† To show how strong this feeling was a hundred years ago in England, we may quote the following passage from Cheselden's *Anat. of the Human Body*, Lond. 1740, 8vo, p. 306: "Some years since a malefactor was pardoned, on condition that he suffered this experiment" [viz., the perforation of the tympanum of the ear, which was, we believe, to have been performed by Cheselden himself], "but he falling ill of a fever, the operation was deferred, during which time there was so great a public clamour raised against it that it was afterwards thought fit to be forbid."

‡ *Χαλεπὸν μὲν ἄνθρωπον ὄντα μὴ διαμαρτάνειν ἐν πολλοῖς, τὰ μὲν ὅλως ἀγνοήσαντα, τὰ δὲ κακῶς κρίναντα, τὰ δὲ ἀμελέστερον γράψαντα.* *De Compos. Medicam. secund. Locos*, lib. ii. c. 1. tom. xii. p. 535.

§ It is not to be found in the British Museum, nor in either of the great libraries at Oxford, nor in the library of the London College of Surgeons, nor in that of the London Medical and Chirurgical Society.

prolapso sic memoraverit." Now the difficulty is, to decide whether Ἡγήτωρ is a proper name, or whether it is merely a sort of honorary title applied to Herophilus, in the same way as we know that Alexander Aphrodisiensis was called, *par excellence*, ὁ ἐξηγήτης, "the Interpreter," on account of the celebrity of his commentaries on Aristotle. In the present instance, Dietz considers Ἡγήτωρ to be the name of one of the physicians of the Herophilean school (Pref. p. viii.), and prints it, accordingly, with a capital letter; while Dr. Marx, as we have seen, takes it in the other way. "Who shall decide when *Doctors* disagree?" and not merely German doctors, but English scholars and doctors also, for it is not by our own unassisted wisdom that we have come to a decision on the subject. The result, however, of our deliberations is, that Dietz is right, and Dr. Marx is wrong, and for these reasons:—We find Ἡγήτωρ mentioned three times by Apollonius (pp. 34, 35, 41), and in only one of these passages is the article prefixed, whereas if it were a designation of Herophilus it could not be used in any instance without it. Then, again, as it is clear * that the writer did not himself belong to the sect of the Herophileans, but was, on the contrary, much opposed to it, it seems hardly probable that he should apply such an honorary title to their founder. It is true that (as far as we are aware,) no mention of a physician of this name occurs elsewhere; but this objection will tell with equal force the other way, for where do we find Herophilus called ἡγήτωρ by any other writer? If it be doubted whether Ἡγήτωρ is ever found as a proper name, we would refer to the new Paris edition of H. Stephens's Greek Thesaurus, where a mathematician of that name is mentioned. If the above remarks be correct, it will appear that Hegetor, and not Herophilus, is the author of the work *De Causis*.† The other works of Herophilus, whose titles are mentioned by Professor Marx (p. 12, seq.), are: *Anatomia; Disquisitiones de Pulsu; Curationes; Commentarius in Hippocratis "Prognostica;" De Oculis; Diætetica;* ‡ *Explicationes Dictionum Exoletarum Hippocratis; Commentarius in Hippocratis "Aphorismos;"* to which may be added one, *Contra Opiniones Vulgares*, Πρὸς τὰς Κοινὰς Δόξας; § and another, *De Arte Obstetricia*, Μαιωτικὸς [Λόγος]. ||

We now come to the most interesting part of Dr. Marx's work, viz., the collection of the medical opinions of Herophilus, as far as they have been handed down to us, and of these we shall try to give an analysis, selecting only those which may be most likely to interest our readers either for their novelty, their truth, or perhaps their strangeness. He owes his principal celebrity (as we have before observed,) to his anatomical researches and discoveries, and several of the names that he gave to dif-

* See Dietz's Preface, p. viii.

† Since writing the above we find that M. Littré, in the introduction to the first volume of his Hippocrates, p. 94, (which we are almost ashamed to confess we had not read sooner,) considers Ἡγήτωρ to be a proper name, and that he is the same person who is mentioned by Galen (*De Dignosc. Puls.* lib. iv. c. 3. tom. viii. p. 955,) in company with several other Alexandrian physicians.

‡ Dr. Marx conjectures (p. 13,) that a work ascribed to "Hierophilus Sophista," entitled *De Diæta et de Facultatibus Alimentorum*, may possibly be a part, or the whole, of this work of Herophilus; the short treatise, however, on this subject by Hierophilus Sophista, which has lately been published by Ideler in the first volume of his *Physici et Medici Græci Minores*, Berol. 1841, 8vo, is evidently the work of a much later author.

§ Quoted by Soranus, *De Arte Obstetr. Morbisque Mulier.* c. 8, p. 21. ed. Dietz, Regim. Pruss. 1838, 8vo.

|| Quoted *ibid.* cc. 48, 93, pp. 100, 211.

ferent parts of the human body remain in common use to this day. He was intimately acquainted with the nervous system (Marx, p. 27, seq.), and seems to have recognised the division of the nerves into those of sensation (*αἰσθητικά*), and those of voluntary motion (*προαιρετικά*); though he included the tendons and ligaments under the common term *νεῦρον*, and called some at least of the nerves by the name of *πόροι*, *meatus*. (Marx, p. 30.) Whether the common point to which the sinuses of the dura mater converge, corresponding with the internal occipital protuberance, to which we now give the name of "Torcular Herophili," be the exact spot which Herophilus himself meant to designate by the term *ληνός*, it is almost impossible to decide: Vesalius confessed the difficulty just three centuries ago, Dr. Marx (p. 28) does the same, and we must follow their example.* He distinguished the cerebrum, *ἐγκέφαλος*, from the cerebellum, *παρεγκεφαλίς*, though he does not seem to have been the first person who did so, as the two words are found in Aristotle.† He placed the seat of the soul (*τὸ τῆς ψυχῆς ἡγεμονικόν*) in the ventricles of the brain (Marx, pp. 29, 37), and thus probably originated the idea which was again brought forward with some modification, towards the end of the last century, by Sömmering.‡ The angular indentation in the posterior part of the medulla oblongata derived the name of "calamus scriptorius" from him (Marx, pp. 5, 30), on account of its similarity (not to the Roman *stilus*, but) to the reed (*κάλαμος*,) used sometimes by the ancients as a pen for writing, of which the best sorts came from Egypt and Cnidos,§ and with which he would naturally therefore be well acquainted. He carefully described the tunics of the eye, and was the first to designate them by peculiar names (Marx, p. 30, seq.), though what is exactly signified by each of these is a minute and difficult question, which perhaps the generality of our readers would not care to see fully discussed in these pages.|| He remarked the difference in the substance of the coats of the veins and arteries, and said that the latter were six times as thick as the former (Marx, p. 31). To the pulmonary artery he gave the name of the *arterial vein* (*φλὲψ ἀρτηριώδης*), as being of an arterial structure, while its contents were venous. He appears to have doubted whether (as was universally believed among the ancients,) the veins were derived from the liver, and to have been very near anticipating Aselli in his discovery of the *lacteals* (Marx, p. 32). The *duodenum* derives its name from him, being called *δωδεκαδάκτυλος ἔκφυσις* (which the Germans translate more literally than the Latins, and call *zwölffingerdarm*) from being about twelve fingers in length.¶ Our readers will perhaps excuse our entering

* Theophilus (*De Corp. Hum. Fabr.* lib. iv. c. 5, § 5,) seems (as is observed in the Oxford edition,) to confound the *torcular* with the *infundibulum.* The passage is not noticed by Professor Marx.

† *Hist. Animal,* lib. i. c. 16, § 3. ed. Bekker, Oxon. See Caspar Hofmann's *Comment. in Galen.* "*De Usu Part.,*" § 636, quoted in the Oxford edit. of Theophilus, p. 134, l. 9.

‡ See his treatise, *Ueber das Organ der Seele,* § 26, 28. Königsberg, 1796, 4to. (Marx, p. 38.)

§ See Pliny, *Hist. Nat.* lib. xvi. c. 64; Martial, *Epigr.* lib. xiv. ep. 38.

|| We agree, however, with Isensee (*Gesch. der Med.* p. 88), in thinking that Dr. Marx is probably wrong if he supposes (as he seems to do, p. 31,) that the *χιτὼν ἀραχνοειδής* in Rufus Ephesius, p. 36, means the *tunica humoris vitrei,* or *hyaloid membrane,* as the *retina* appears to us to answer best to the description. Any one who is curious on the subject may refer to the notes in the Oxf. edit. of Theophilus, on p. 159, l. 6, and p. 164, l. 7.

¶ See Theophilus, *De Corp. Hum. Fabr.* lib. ii. c. 7, § 10; a passage which appears to have escaped Professor Marx's search.

minutely into the opinions of Herophilus respecting the organs of generation, on account of the uncertainty that prevails concerning the meaning of some of the terms in use among the ancients, an uncertainty which (it may be remarked) is increased in each particular instance, by our not feeling quite sure whether it arises from *their* imperfect or erroneous description of the part, or our own dulness. The female organs he is expressly said by Galen to have studied in the human subject, and accordingly he particularly noticed the different appearance presented by the *os uteri* in its natural state, during menstruation, and during pregnancy.* He was also acquainted with the *Fallopian tubes*, and the *ovaries*, which latter organs (in order probably the better to preserve the analogy between the two sexes) he called *the testicles* (Marx, p. 36), a name which, we believe, continued in use till quite modern times. His opinion concerning the *umbilical vein* and *arteries* (which we find in Soranus) † seems to contain a singular mixture of truth and error. He followed Aristotle, ‡ in supposing that there were *two* umbilical veins as well as two arteries, § composing the placenta; || of which the former terminated in the vena cava, and the latter in the trachea, after first turning off to each side along the bladder. ¶ Whether, in this case, Herophilus confused the trachea with the aorta, or whether Soranus (or his transcriber) has mistaken his opinion, seems doubtful; though, if the blunder really be Herophilus's own, it is a strange instance of carelessness or ignorance, as Aristotle expressly says that the umbilical arteries join the aorta, where it subdivides into two branches.**

The opinions of Herophilus on pathology, dietetics, diagnosis, therapeutics, materia medica, surgery, and midwifery, are collected by Dr. Marx with equal care, but need not be here analysed so minutely, as we have already stated that his fame rests chiefly on his superior knowledge of anatomy and physiology. He was, as might be expected, a favourer of the humoral pathology (Marx, p. 43); he is accused by Galen of neglecting to inquire into the causes why, in some cases of paralysis, sensation only is injured, in others motion only, and in others, again, both sensation and motion, though he was aware of the fact (Marx, pp. 44, 57); he appears to have given great attention to the pulse, of which he made several subtle and minute divisions, though he has not the honour of being the first

* It would seem, from his quoting Soranus through the medium of the Latin translation of Oribasius (p. 35) that Dietz's Greek edition of the complete work had never fallen in the way of Professor Marx, which we regret the more, as he would have been able to have added from it some interesting notices of the opinions of Herophilus on different subjects connected with midwifery. See pp. 69, 99, 100, 123, 210, 211, &c.

† *De Arte Obstetr.* c. 22, p. 69.

‡ *Histor. Animal.* lib. vii. c. 8, § 6.

§ This opinion seems to have prevailed almost universally among the ancients (See Rufus Ephes. *De Appellat. Part. Corp. Hum.* p. 45; Galen, *De Usu Part.* lib. xv. c. 5. tom. iv. p. 231; Theophilus, *De Corp. Hum. Fabr.* lib. v. c. 19, § 4), and evidently arose from the dissection of animals, many of which have two umbilical veins. See Haller, *Physiol.* lib. xxix. sect. 3. § 18, tom. viii. p. 220.

|| Τὸ *χορίον*, under which name both the *placenta* and the *chorion* were formerly comprehended. See Casper Hofmann's *Comment. in Galen.* "*De Usu Part.*" § 1068; Haller, *Physiol.* lib. xxix. sect. 3. § 5, 21. tom. viii. pp. 187, 226.

¶ *Ἡρόφιλος δὲ τὰς φλέβας μὲν εἰς τὴν κοίλην φλέβα, ἀρτηρίας δὲ εἰς τὴν τραχεῖαν ἀρτηρίαν, τὴν παρατείνουσαν τοῖς σπονδύλοις· πρὸ δὲ τῆς εἰς αὐτὴν ἐμφύσεως παρὰ τὴν κύστιν αὐτὰς πλαγιοφορεῖσθαι παρ' ἑκατέρας πλευράς.*

** *Αἱ δὲ δύο [φλέβες] πρὸς τὴν ἀορτὴν, ᾗ σχίζεται καὶ γίγνεται ἡ ἀορτὴ δύο ἐκ μιᾶς.* *Hist. Animal.* lib. vii. c. 8, § 6.

person who discovered its value as a symptom of disease. (Marx, p. 46, &c.) Perhaps the weakest point in Herophilus was his pharmaceutical practice, as he seems to have been one of the earliest physicians who administered large doses of hellebore, and other drastic purgatives,* and who (on the principle that compound diseases require compound medicines,) began that strange system of heterogeneous mixtures, some of which have only lately been expelled from our own pharmacopœia, and which still keep their place on the continent.† The principal part of Dr. Marx's account of the surgical practice of Herophilus consists of rather a long fragment on the mode of reducing a luxation of the femur, taken from the work *De Causis* (Marx, pp. 58, 59), which, however, (if what we have said above be correct,) was written by Hegetor, and not by Herophilus. It is curious to find him stating that round ulcers are more difficult to heal than others (Marx, p. 59); an opinion which, like many similar modern discoveries, seems to have been neglected and forgotten for centuries, to be again brought to light in our own days. With respect to his practice in obstetric medicine, we learn incidentally that in certain cases he considered the sacrifice of the fœtus allowable (Marx, p. 60). This is brought against him as a charge by Tertullian, and certainly (from what we know of the lax opinions of the ancients on this point),‡ we must confess that the instrument he describes may very likely have been much abused; we know also that the question cannot be even yet considered as unanimously settled; but still we cannot but think that Herophilus was clearly right in theory, however much his example may formerly have been followed wantonly and unnecessarily, and that, when argued on religious and moral grounds, this question becomes one of the simplest and plainest in the code of medical ethics.§

Such is the imperfect abstract which we have been able to make of the life and opinions of this celebrated man, which is derived, be it remembered, not from the perusal of one or more of his own complete works, but from extracts and allusions found in other authors, which we may

* See Isensee, *Gesch. der Med.* p. 91.

† In the London Pharmacopœia both the celebrated *Mithridatium* and the *Theriaca Andromachi* were retained, certainly as late as the year 1771, and the edition of 1788 is the earliest in which we have noticed their omission. In the last edition of the Paris Pharmacopœia (4to, 1837,) the *Theriaca* still appears in full glory.

‡ Thus, though in the Hippocratic "Oath" it is declared to be unlawful to produce abortion, in another part of the Hippocratic Collection (*De Nat. Pueri*, tom. i. p. 385,) we meet with the details of a case in which this was done, related without the smallest appearance of compunction: an inconsistency, by the way, which those commentators who suppose the two works to be written by the same person have been much puzzled to reconcile or account for. See Joannes Alexandr. in Dietz's *Scholia in Hippocr. et Gal.* tom. ii. p. 216.

§ Professor Marx supposes (pp. 7, 60,) that it was Herophilus who instructed Agnodice in midwifery, and that therefore, in Hyginus (*Fab.* 274, p. 201), instead of "*Hierophilo* cuidam" we must read "*Herophilo* cuidam." The conjecture is certainly very tempting, and has occurred to so many of his biographers, that we are almost afraid to start any objections; but still we would venture to suggest,—1. Whether the whole story (if it is to be believed at all on the authority merely of Hyginus,) does not seem to belong to the sixth or fifth century before Christ, rather than to the fourth or third? 2. Whether the emendation is not *so very* easy and obvious, that (according to a well-known law in criticism) the more difficult reading ("*Hierophilo,*") is on that very account the more likely to be the true one? 3. Whether it is known that Herophilus was ever at Athens, or Agnodice at Alexandria? 4. Whether it is likely that Hyginus would call so universally famous a physician as Herophilus, simply "*one* Herophilus" (Herophilus *quidam*)?

fairly presume to be in some cases given imperfectly, in others, perhaps, to have been mistaken or even misrepresented; and if we do not think him quite so near to perfection as Professor Marx does, p. 60, (since we all know that biographers often entertain an amiable prejudice in favour of their hero,) still we are fully inclined to admit that he will always rank among those few whose names are preserved from oblivion, not by the mere accident of their writings having escaped destruction, but on account of their successful endeavours to promote the advancement of science, and to add something to the amount of human knowledge, by finding a few shells and pebbles on the shore of the Ocean of Truth.

With respect to the diligence and ability with which the work is executed, we have already expressed our opinion; and though in some points we have differed from the author, and have explained our reasons for doing so, yet Dr. Marx (who is himself a reviewer,) will easily conceive that we should not have taken the trouble to do this in the case of a work of which we did not think highly.* In conclusion, we would venture to suggest to Dr. Marx himself the execution of another work which he has expressed a wish (p. 16,) to see undertaken by some one fitted for the task, a work of similar plan with the present, to which it would form the most suitable companion; we mean an account of the life, writings, and opinions of that remarkable man, the contemporary of Herophilus at the court of Ptolemy, with whose name his own is still commonly connected, his equal in reputation during life, and his rival in posthumous renown—ERASISTRATUS.

* We are sorry to be obliged to add, that the printer has not done justice to the work, as, for so small a book, there are certainly an unusual number of typographical errors, and some of considerable importance.

